# Heat Inactivation of Nipah Virus for Downstream Single-Cell RNA Sequencing Does Not Interfere with Sample Quality

**DOI:** 10.3390/pathogens13010062

**Published:** 2024-01-09

**Authors:** Adam J. Hume, Judith Olejnik, Mitchell R. White, Jessie Huang, Jacquelyn Turcinovic, Baylee Heiden, Pushpinder S. Bawa, Christopher J. Williams, Nickolas G. Gorham, Yuriy O. Alekseyev, John H. Connor, Darrell N. Kotton, Elke Mühlberger

**Affiliations:** 1 Department of Virology, Immunology and Microbiology, Chobanian & Avedisian School of Medicine, Boston University, Boston, MA 02118, USA; hume@bu.edu (A.J.H.); jolejnik@bu.edu (J.O.); mitchw@bu.edu (M.R.W.); jturcino@bu.edu (J.T.); bheiden@bu.edu (B.H.); jhconnor@bu.edu (J.H.C.); 2 National Emerging Infectious Diseases Laboratories, Boston University, Boston, MA 02218, USA; 3 Center for Regenerative Medicine of Boston University and Boston Medical Center, Boston, MA 02118, USA; hjess@bu.edu (J.H.); bpushpin@bu.edu (P.S.B.); dkotton@bu.edu (D.N.K.); 4 The Pulmonary Center and Department of Medicine, Chobanian & Avedisian School of Medicine, Boston University, Boston, MA 02118, USA; 5 Department of Medicine, Single Cell Sequencing Core Facility, Chobanian & Avedisian School of Medicine, Boston University, Boston, MA 02118, USA; cjwill12@bu.edu; 6 Microarray and Sequencing Resource Core Facility, Chobanian & Avedisian School of Medicine, Boston University, Boston, MA 02118, USA; ngorham@bu.edu; 7 Department of Pathology and Laboratory Medicine, Chobanian & Avedisian School of Medicine, Boston University, Boston, MA 02118, USA; yurik@bu.edu

**Keywords:** Nipah virus, single-cell RNA sequencing, scRNA-seq, 10x Genomics, heat inactivation, BSL-4 pathogens, VSV, iPSC-derived alveolar type 2 cells, nonsegmented negative sense RNA viruses

## Abstract

Single-cell RNA sequencing (scRNA-seq) technologies are instrumental to improving our understanding of virus–host interactions in cell culture infection studies and complex biological systems because they allow separating the transcriptional signatures of infected versus non-infected bystander cells. A drawback of using biosafety level (BSL) 4 pathogens is that protocols are typically developed without consideration of virus inactivation during the procedure. To ensure complete inactivation of virus-containing samples for downstream analyses, an adaptation of the workflow is needed. Focusing on a commercially available microfluidic partitioning scRNA-seq platform to prepare samples for scRNA-seq, we tested various chemical and physical components of the platform for their ability to inactivate Nipah virus (NiV), a BSL-4 pathogen that belongs to the group of nonsegmented negative-sense RNA viruses. The only step of the standard protocol that led to NiV inactivation was a 5 min incubation at 85 °C. To comply with the more stringent biosafety requirements for BSL-4-derived samples, we included an additional heat step after cDNA synthesis. This step alone was sufficient to inactivate NiV-containing samples, adding to the necessary inactivation redundancy. Importantly, the additional heat step did not affect sample quality or downstream scRNA-seq results.

## 1. Introduction

The development of high-throughput sequencing technologies has made it possible to determine the transcriptomic landscape at a single cell level. As for viral infection studies, single-cell RNA sequencing (scRNA-seq) does not only allow for discrimination between infected and non-infected bystander cells, but it also provides insight into cell type-specific responses in tissue culture as well as complex organs and immune systems [1,2,3]. However, the more stringent biosafety requirements using highly pathogenic viruses have hindered scRNA-seq analysis in studies involving viruses handled at the highest biosafety level, BSL-4. To protect the environment and the researchers handling the material, samples that contain highly pathogenic viruses must be completely inactivated before they can be transferred to lower biosafety levels for downstream analyses. In addition, almost all BSL-4 viruses are classified as Select Agents by the United States Federal Select Agent Program (FSAP), which is jointly composed of the Centers for Disease Control and Prevention/Division of Select Agents and Toxins and the Animal and Plant Health Inspection Service/Division of Agricultural Select Agents and Toxins. FSAP regulations require that inactivation procedures must be validated or verified in-house (at each research institution) to confirm complete inactivation [4]. While some scRNA-seq procedures include the treatment of cells with fixatives that might lead to virus inactivation, such as 80% methanol, acetone:methanol, 4% paraformaldehyde, or 3% glyoxal [5,6,7,8], a careful adaptation to BSL-4 requirements would be necessary (e.g., longer incubation times) which could affect the quality of the cellular RNA. We have previously shown that inactivation of BSL-4 samples using heat did not compromise the quality of cellular RNA for downstream high-throughput sequencing of low cell numbers [9]. The advantage of heat inactivation is that it is easily adaptable to various conditions, including cell numbers, viral load, temperature, and incubation time.

**Figure 1:**
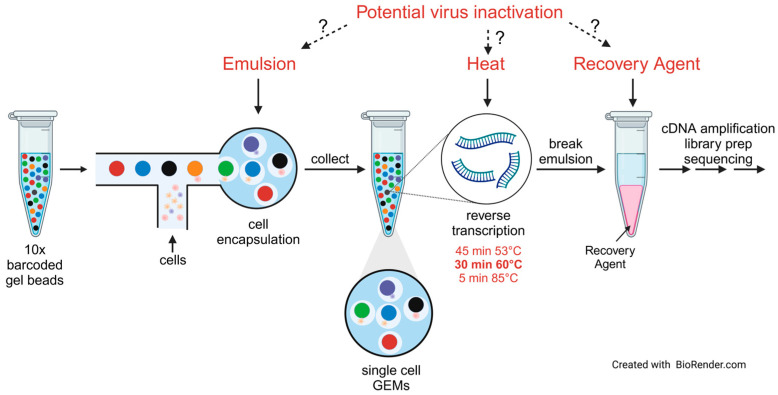
Scheme of the 10x Genomics Chromium single-cell sequencing platform. Potential inactivation steps are indicated in red. An additional heat inactivation step after cDNA synthesis at 53 °C was added (30 min at 60 °C, in bold) that is not part of the original protocol.

Here, we describe a heat-based inactivation protocol for scRNA-seq analysis of BSL-4 samples using the Chromium Next GEM Single Cell 3ʹ Reagent Kits v3.1 (10x Genomics, Pleasanton, CA, USA). This sequencing platform is based on the formation of gel beads in emulsion (GEMs) on the Chromium microfluidic chip (10x Genomics, Pleasanton, CA, USA) (Figure 1). Each GEM contains a single cell, a gel bead containing barcoded oligonucleotides, and reverse transcription reagents. Within the GEM, the cell is lysed, and the gel bead is dissolved to release identically barcoded oligonucleotides for cDNA synthesis. Reverse transcription of polyadenylated mRNA occurs at 53 °C for 45 min, followed by a 5 min incubation at 85 °C. After this initial cDNA synthesis step, a Recovery Agent is added to the sample for demulsification. The aqueous phase is then used for cDNA library preparation, which desirably should be performed outside maximum containment.

For our studies, we used *Henipavirus nipahense* as our prototype virus. Nipah virus (NiV) belongs to the family *Paramyxoviridae* and has a nonsegmented negative-sense RNA genome. NiV infection in humans results in severe respiratory and neurological disease with high case fatality rates [10,11]. Due to its high pathogenicity, its transmissibility, and the lack of approved treatment options or vaccines, NiV is classified as a BSL-4 pathogen and Select Agent. NiV infects a variety of cell types and organs, inducing distinct host response signatures [12,13]. Single-cell analysis of NiV-infected tissue would be highly informative toward a better understanding of NiV pathogenesis and the development of antiviral countermeasures. In addition, inactivation procedures that render NiV non-infectious have a high likelihood to also be effective against other BSL-4 negative-sense RNA viruses, including filoviruses, arenaviruses, and nairoviruses [14,15].

In this study, we tested various steps of the Chromium protocol for their ability to inactivate negative-sense RNA viruses. We show that the reagents used for GEM generation and demulsification are not sufficient for chemical virus inactivation. In contrast, the 5 min at 85 °C incubation step alone following cDNA synthesis was sufficient to inactivate NiV under the conditions described below. For safety reasons, we added an additional heat inactivation step (30 min at 60 °C) following the most critical step of the scRNA-seq procedure, reverse transcription of the cellular mRNA. This assures that single-cell transcriptomes are not compromised by the inactivation step, which could lead to the loss of transcripts and amplification of the compromised transcriptome during downstream procedures [16,17,18,19,20]. We also show that the addition of the 60 °C inactivation step did not compromise the quality of scRNA-seq results.

## 2. Materials and Methods

### 2.1. Biosafety Statement

All work with NiV was performed in the BSL-4 facility of Boston University’s National Emerging Infectious Diseases Laboratories (NEIDL) following approved standard operating procedures in compliance with local and national regulations pertaining to handling BSL-4 pathogens and Select Agents.

### 2.2. Immortalized Cells

Vero E6 cells (African green monkey kidney cells; ATCC CRL-1586) were maintained in Dulbecco’s modified Eagle medium (DMEM; Gibco/Thermo Fisher Scientific, Waltham, MA, USA) supplemented with 2 mM L-glutamine (Thermo Fisher Scientific, Waltham, MA, USA), 100 μg/mL Primocin (Invivogen, San Diego, CA, USA), and 10% fetal bovine serum (FBS; R&D Systems, Minneapolis, MN, USA). Cells were grown at 37 °C and 5% CO_2_.

### 2.3. Induced Pluripotent Stem Cell Directed Differentiation of Alveolar Type 2 Cells

All experiments involving the differentiation of human pluripotent stem cell lines were performed with the approval of the Institutional Review Board of Boston University (protocol H33122). The SPC2 induced pluripotent stem cell (iPSC) line carrying an SFTPC^tdTomato^ reporter was obtained from our previous studies [21].

The human iPSC line, clone SPC-ST-B2, was differentiated into iPSC-derived alveolar-epithelial-type-2-like (iAT2) cells in 3D Matrigel cultures as previously described [22]. PSC-directed differentiation via definitive endoderm into NKX2-1 lung progenitors was performed as follows: cells maintained in mTeSR1 media (StemCell Technologies, Vancouver, BC, Canada) were differentiated into definitive endoderm using the STEMdiff Definitive Endoderm Kit (StemCell Technologies, Vancouver, BC, Canada). After the endoderm-induction stage, cells were dissociated, passaged into 6-well plates pre-coated with growth factor reduced Matrigel, and differentiated as previously described in detail [23,24,25]. To establish pure iAT2 cell cultures, cells were sorted using flow cytometry to isolate SFTPC^tdTomato+^ cells on day 44 of differentiation. iAT2 cells were then maintained via serial passaging as self-renewing monolayered epithelial spheres (‘‘alveolospheres’’) by plating in Matrigel droplets at a density of 400 cells/µL with refeeding every other day in CK+DCI medium [26]. iAT2 cell culture quality and purity were monitored at each Passage using flow cytometry, with >80% of cells expressing SFTPC^tdTomato^ over time, as we have previously detailed [26,27].

Air–liquid interface (ALI) cultures were prepared as described before [24,25]. Briefly, Matrigel droplets containing iAT2 cells as 3D sphere cultures were dissolved in 2 mg/mL dispase Sigma Aldrich, San Luis, MO, USA), and alveolospheres were dissociated in 0.05% trypsin (Thermo Fisher Scientific, Waltham, MA, USA) to generate a single-cell suspension. 6.5 mm transwell inserts (Corning, Glendale, AZ, USA) were coated with diluted Matrigel according to the manufacturer’s instructions. Cells were plated on the transwells at a density of 5.2 × 10^5^ live cells per cm^2^ in 100 µL of CK+DCI with 10 µM Rho-associated kinase inhibitor (“Y”; Sigma Y-27632). 600 µL of CK+DCI+Y was added to the basolateral compartment. 24 h after plating, basolateral media was refreshed to CK+DCI+Y. 48 h after plating, apical media was aspirated to initiate air–liquid interface culture. 72 h after plating, basolateral media was exchanged with CK+DCI to remove the rho-associated kinase inhibitor. Basolateral media was changed three times per week thereafter.

### 2.4. Virus Propagation

NiV (strain Bangladesh, GenBank number NC_002728.1) was kindly provided by H. Feldmann, NIH NIAID Rocky Mountain Laboratories, Hamilton, MT. Recombinant vesicular stomatitis virus (VSV) expressing Ebola virus (EBOV) glycoprotein (GP) in place of VSV G and expressing GFP as an additional open reading frame (rVSV-GP_EBOV_-GFP) was kindly provided by A. Marzi, NIH NIAID Rocky Mountain Laboratories, Hamilton MT [28]. All viruses were propagated in Vero E6 cells in DMEM supplemented with 2 mM L-glutamine, 100 μg/mL Primocin, and 2% FBS. Virus titers were determined in Vero E6 cells by tissue culture infectious dose 50 (TCID_50_) assay using the Spearman and Kärber algorithm [22,29].

### 2.5. Testing Virus Inactivation by Recovery Agent

To determine if Recovery Agent (10x Genomics, Pleasanton, CA, USA) is cytopathogenic, 125 µL of Recovery Agent was added to 1 mL PBS and then transferred to a T75 flask of Vero E6 cells. The inoculum was removed after 1 h and replaced with 15 mL DMEM supplemented with L-glutamine, Primocin, and 2% FBS. The cells were incubated overnight and monitored for cytopathic effect (CPE).

To remove cytotoxic substances contained within Recovery Agent, we used Bio-Beads SM2 resin (Bio-Rad, Hercules, CA, USA) in a batch protocol. 0.5 g Bio-Beads SM2 resin was washed in 3 mL of methanol, then in 3 mL of H_2_O, and finally in 3 mL of PBS. The Bio-Beads were stored in 1 mL of PBS at 4 °C for up to 1 week or until further use. To test whether Bio-Beads could be used to remove cytotoxic Recovery Agent from a solution, 125 µL of Recovery Agent was added to 0.2 g Bio-Beads in 1 mL PBS, and the solution was mixed on a rotary platform for 15 min at room temperature. After a centrifugation step at 27 rcf for 2 min in a microcentrifuge, the supernatant was transferred onto Vero E6 cells seeded in T75 flasks. After a 1 h incubation at 37 °C, the cell supernatant was removed and replaced by 15 mL DMEM supplemented with L-glutamine, Primocin, and 2% FBS. The cells were incubated overnight at 37 °C and 5% CO_2_ and monitored for signs of CPE.

To test if Recovery Agent had antiviral properties, 125 µL rVSV-GP_EBOV_-GFP stock solution (2.3 × 10^8^ TCID_50_ units/mL) or 125 µL PBS was mixed with 125 µL Recovery Agent. To test if the emulsion (10x Genomics, Pleasanton, CA, USA) used for GEM generation would influence any potential antiviral effects of Recovery Agent, 125 µL rVSV-GP_EBOV_-GFP stock solution was mixed with 125 µL emulsion, and 125 µL Recovery Agent was added. Following the Chromium platform protocol, the biphasic solutions were incubated for 2 min at room temperature without mixing. The solutions were then transferred to tubes containing 0.2 g Bio-Beads SM-2 resin in 1 mL PBS (prepared as described above) and mixed by pipetting followed by gentle mixing for 15 min at room temperature on a rotating mixer. After a 2-min centrifugation at 27 rcf, the supernatants were used to infect Vero E6 cells seeded in T75 flasks. The cells were incubated for 1 h at 37 °C, the inoculum was removed and replaced with 15 mL DMEM supplemented with L-glutamine, Primocin, and 2% FBS, and the cells were incubated overnight at 37 °C and 5% CO_2_. Viral infection was determined by visualizing GFP expression.

### 2.6. Testing Virus Inactivation Using Heat

The MiniAmp™ Thermal Cycler, catalog number A37834 (Applied Biosystems, Waltham, MA, USA), was used for this study. In addition to the internal thermometer of the thermal cycler, block temperature was measured using the digital Fluke T3000 FC Wireless K-Type Temperature Module (Fluke Corporation, Everett, WA, USA) as an external thermometer. This module can record up to 65,000 temperature readings with a resolution of 0.1 °C and an accuracy of ±[0.5% + 0.5 °C]. The temperature sensor (thin wire) was placed in a test tube filled with 125 μL of water, and the tube was placed in the thermal cycler. Due to the thin wire sensor, it was possible to close the lid of the thermal cycler.

To test the ability of heat to inactivate NiV for scRNA-seq analysis, 6 × 10^4^ Vero E6 cells per well of a 48-well plate were mock-infected or infected with NiV at a multiplicity of infection (MOI) of 10 TCID_50_ units per cell. One day post-infection (dpi), the cells were washed once with PBS, detached, and transferred into tubes. Cells were spun down, resuspended into 125 μL of PBS, and transferred into MicroAmp 8-tube strips (Applied Biosystems, Waltham, MA, USA). The mock-infected control was used to determine cell numbers (1 × 10^5^ cells after one dpi). The tubes containing the samples were placed in the thermal cycler and exposed to 53 °C for 45 min, 60 °C for 30 min, 85 °C for 5 min, or a combination of these conditions. Control samples were incubated at room temperature for 80 min.

To determine the initial infection rates, additional wells in the 48-well plate were infected in parallel with NiV at the same MOI (MOI = 10). These cells were fixed at the time of virus inactivation (1 dpi) for at least 6 h with 10% formalin and analyzed for the presence of NiV using immunofluorescence analysis, as described in Section 2.8.

The cells incubated at various conditions were then used to infect 4 × 10^6^ Vero E6 cells seeded in T75 flasks. For the NiV-challenge sample, NiV was mixed with non-infected cells that had been incubated at 53 °C for 45 min, followed by an incubation at 60 °C for 30 min and an incubation at 85 °C for 5 min. This mixture was used to infect cells at an MOI of 0.01.

The cells from the test infection were incubated for 4 days and checked for signs of CPE. At 4 dpi, cell supernatants were passaged onto fresh cells to further amplify the virus. To do this, cell supernatants were clarified using low-speed centrifugation, and the entire supernatant was used to infect Vero E6 cells seeded in T75 flasks.

On day 4 post-infection, cell supernatants from the 1st Passage were clarified using low-speed centrifugation, and 0.2 mL was used to infect Vero E6 cells seeded in a 96-well plate. Two days post-infection, the cells were fixed with 10% formalin (LabChem, Zelienople, PA, USA) and subjected to immunofluorescence analysis using NiV-specific antibodies.

### 2.7. Testing Virus Inactivation following GEM Generation

To investigate if the generation of GEMs with the Chromium instrument affected virus inactivation, we used rVSV-GP_EBOV_-GFP as our test virus. 8 × 10^5^ VeroE6 cells per well of a 6-well plate were mock-infected or infected with rVSV-GP_EBOV_-GFP at an MOI of 1. 18 h post-infection, the cells were washed with PBS, detached by trypsinization, and transferred into tubes. The cells were prepared for GEM generation according to the 10x Genomics Cell Preparation Guide. Briefly, infected cells were pelleted using centrifugation at 150 rcf for 3 min at 4 °C. Supernatant was discarded, and the cell pellet was resuspended using pipetting in 1 mL DMEM supplemented with L-glutamine and 7% FBS (DMEM + 7%FBS). The cells were counted manually via hemocytometer, and the percentage of live cells was calculated. Centrifugation, resuspension, and counting were repeated once to obtain >90% viability and to concentrate 1 × 10^5^ cells into 70 µL cell culture media.

GEMs were generated according to the 10x Genomics Chromium Next GEM User Guide v3.1 using training reagents (10x Genomics, Pleasanton, CA, USA) such that each well in the chip contained 1 × 10^5^ live cells. Following GEM generation on the Chromium instrument, GEMs were processed without cDNA synthesis (no exposure to heat) or subjected to cDNA synthesis for 45 min at 53 °C followed by the additional heat step for 30 min at 60 °C and a 5 min incubation at 85 °C. As a positive control for heat inactivation, 1 × 10^5^ rVSV-GP_EBOV_-GFP-infected cells in 125 µL DMEM + 7% FBS were incubated for 45 min at 53 °C, 30 min at 60 °C and 5 min at 85 °C. Additional controls included 1 × 10^5^ cells untreated mock-infected or rVSV-GP_EBOV_-GFP-infected cells in 125 µL DMEM + 7% FBS. Samples were transferred onto 4 × 10^6^ VeroE6 cells seeded in T75 flasks, and images were taken at 1 dpi and 2 dpi on a Nikon TS100 inverted microscope to assess infection via GFP expression.

### 2.8. Immunofluorescence Analysis

2 × 10^4^ Vero E6 cells seeded in 96-well plates were infected with 200 µL of cell supernatant (3 wells per test sample), and three 1:10 serial dilutions were performed per well. Infected cells were incubated for the indicated times at 37 °C and fixed with 10% formalin. The fixed cells were permeabilized with 0.1% Triton X100 (Boston Bioproducts, Milford, MA, USA) for 5–10 min at room temperature, incubated in 0.1 M glycine (Boston Bioproducts, Milford, MA, USA) for 5 min at room temperature, and subsequently incubated in 5% goat serum (Jackson ImmunoResearch, West Grove, PA, USA) for 20–60 min at room temperature. After each step, the cells were washed three times in PBS. The cells were incubated overnight at 4 °C with polyclonal anti-NiV hyperimmune mouse ascitic fluid (BEI; 1:200 dilution in 5% goat serum), washed four times in PBS and incubated with a chicken anti-mouse antibody conjugated with AlexaFluor488 (Invitrogen; 1:200 dilution in 5% goat serum) plus 4′,6-diamidino-2-phenylindole (DAPI; Sigma-Aldrich at 200 ng/mL for nuclei staining) for 1 h at room temperature. Images were acquired using a Nikon TS100 Eclipse microscope and Nikon DS Qi1Mc camera with NIS Elements F software or with a Nikon Eclipse Ti2 microscope with Photometrics Prime BSI camera and NIS Elements AR software.

### 2.9. Limit of Detection Analysis

To assess the sensitivity of our experimental design, a limit of detection analysis was included. 2 × 10^6^ Vero E6 cells were seeded in T75 flasks and one day later, infected with 1, 10, or 100 TCID_50_ units of NiV stock diluted in cell culture medium supplemented with 2% FBS. The cells were monitored for CPE. 4 dpi, the entirety of the supernatants was clarified using low-speed centrifugation and transferred onto Vero E6 cells seeded in T75 flasks. 4 dpi, 0.2 mL of each of the supernatants was used to infect Vero E6 cells seeded in 96-well plates. After 2 days of infection, the cells were fixed with 10% formalin and used for immunofluorescence analysis. The limit of detection analysis was performed in two independent experiments.

### 2.10. Single-Cell RNA Sequencing

Human iPSC-derived alveolar epithelial type 2 cells (iAT2s) at day 216 of differentiation were seeded at 230,000 cells per Transwell insert, and the air–liquid interface was established as described above. At 14 days post-seeding, iAT2s were washed three times with PBS and dissociated using Accutase cell detachment solution (Stemcell Technologies, Cambridge, MA, USA) for 20 min at room temperature and resuspended in 1% FBS in PBS with 10 µM Y-27632 and calcein blue. Live singlets were sorted using the MoFlo Astrios cell sorter and loaded onto the 10x Genomics chip at 9 × 10^5^ cells/mL with >90% viability for a targeted recovery of 1000 cells using 10x Genomics Single Cell 3′ v3.1 Dual-Index kit per the manufacturer’s guidance. After the 10x Genomics Chromium Controller run, the emulsion from the recovery wells of the chip was transferred to a PCR strip. The PCR strip was loaded into a thermal cycler for cDNA synthesis with the following parameters: 45 min at 53 °C, 5 min at 85 °C, hold at 4 °C. Alternatively, samples undergoing an additional secondary heat step were loaded with the following parameters: 45 min at 53 °C, 30 min at 60 °C, 5 min at 85 °C, hold at 4 °C. Tubes were not opened during or after heat inactivation. Samples were subsequently stored at −20 °C overnight. The next day, samples were thawed at room temperature before adding a Recovery Agent. Since the removal of inactivated samples from the BSL-4 laboratory involves a decontamination step of the tubes in 5% MicroChem Plus (National Chemical Laboratories Inc., Philadelphia, PA, USA) within a dunk tank for at least 10 min, we mimicked this step by placing one sample in a secondary container of chilled 5% Microchem Plus. After a 10-min incubation time in 5% Microchem Plus, the sample was removed from the container and allowed to thaw at room temperature prior to the addition of Recovery Agent. After this step, the cDNA libraries were generated according to the 10x Genomics protocol, and their size distribution and molarity were assessed via the Bioanalyzer High Sensitivity DNA Assay (Agilent Technologies, Lexington, MA, USA). All cDNA libraries were sequenced on an Illumina NextSeq 2000 instrument with 850 pM input and 2% PhiX control library spike-in using P1 100 cycle flowcell (Illumina, San Diago, CA, USA).

### 2.11. scRNA-seq Data Analysis

Sequencing files were mapped to the human genome reference (GRCh37) supplemented with td^Tomato^ sequences using CellRanger v3.0.2. Seurat v3.2.3 [30] was used for downstream analysis and quality control. After inspection of the quality control metrics, cells with 15% to 35% mitochondrial content and <800 detected genes were excluded from downstream analyses. In addition, doublets were also excluded for downstream analysis. We normalized and scaled the unique molecular identifier (UMI) counts using the regularized negative binomial regression (SCTransform) [31]. Following the standard procedure in Seurat’s pipeline, we performed linear dimensionality reduction (principal component analysis) and used the top 20 principal components to compute the unsupervised Uniform Manifold Approximation and Projection (UMAP) [32]. For clustering of the cells, we used the Louvain algorithm [33], which was computed at a range of resolutions from 1.5 to 0.05 (more to fewer clusters). Populations were annotated using Louvain Clustering at a resolution of 0.05. Cell cycle scores and classifications were performed using Seurat’s cell-cycle scoring and regression method [34]. Cluster-specific genes were calculated using the MAST framework in Seurat wrapper [35].

## 3. Results

### 3.1. Chromium scRNA-seq Workflow and Identification of Potential Steps for Virus Inactivation

There are a couple of steps in the 10x Genomics Chromium scRNA-seq protocol that might allow for virus inactivation. First, inactivation could be performed prior to GEM formation by cell fixation [6,36]. However, inactivating BSL-4 agents is based on an abundance of caution, including long incubation times for fixates to ensure complete inactivation, which might compromise RNA quality. Secondly, virus inactivation could occur during GEM formation prior to reverse transcription via chemical inactivation. We have, therefore, tested the ability of the GEM-forming emulsion to inactivate negative-sense RNA viruses. The next step for potential inactivation is reverse transcription, which includes two heat steps that might lead to virus inactivation. We have tested the heat steps already included in the protocol and added a third heat step to comply with an important concept of BSL-4 inactivation—redundancy. Finally, the Recovery Agent could potentially destroy enveloped viruses, leading to virus inactivation. We have tested this as well in the presented study (Figure 1).

### 3.2. Neither Emulsion nor Recovery Agent Have Antiviral Activity

We first tested the ability of Recovery Agent in the presence and absence of emulsion to inactivate enveloped, nonsegmented negative-sense RNA viruses, the rationale being that adding Recovery Agent is already part of the Chromium platform sequencing protocol and would not compromise sample quality. Emulsion is added to the cells for GEM formation. Following the cDNA synthesis and heat inactivation steps, Recovery Agent is used to break single-cell emulsions and separate the aqueous phase containing the cDNA library from the organic phase containing the partitioning oil and Recovery Agent. To avoid unnecessary time in the BSL-4 laboratory, we tested Recovery Agent using recombinant VSV as a BSL-2 surrogate virus for NiV. Both VSV and NiV belong to the group of nonsegmented negative-sense RNA viruses and are structurally related. The virus used for these studies, rVSV-GP_EBOV_-GFP, expresses EBOV GP as the viral surface protein and GFP as a fluorescent reporter gene [28].

**Figure 2:**
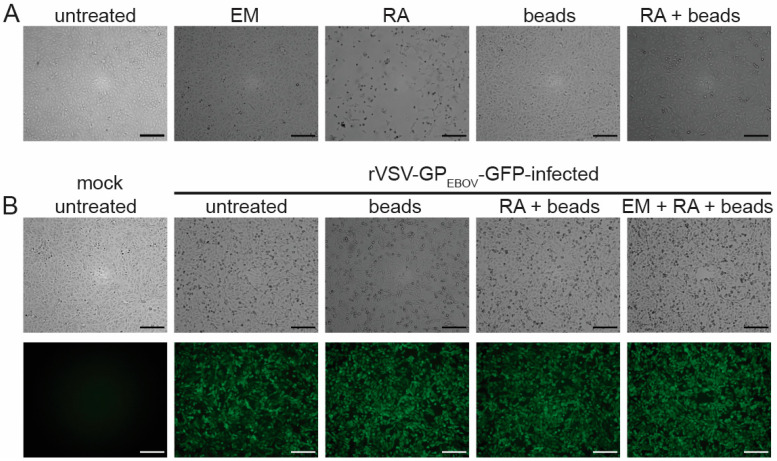
Neither Recovery Agent nor emulsion of the Chromium Next GEM Single Cell Kit (10x Genomics, Pleasanton, CA, USA) displays antiviral activity against rVSV-GP_EBOV_-GFP. (**A**) Vero E6 cells seeded in T75 flasks were left untreated or were treated with emulsion (EM), Recovery Agent (RA), Bio-Beads (beads), or Recovery Agent treated with Bio-Beads (RA + beads). Cells treated with RA showed a clear CPE. None of the other treatments induced CPE. (**B**) 125 µL of rVSV-GP_EBOV_-GFP solution were mixed with 125 µL of either PBS (untreated), Bio-Beads (beads), Recovery Agent treated with Bio-Beads (RA + beads), or emulsion mixed with Recovery Agent treated with Bio-Beads (EM + RA + beads) and used to inoculate Vero E6 cells seeded in T75 flasks. At 1 dpi, virus infection was monitored using GFP expression. None of the treatments led to virus inactivation. Images represent two independent experiments with similar outcomes. Scale bars = 200 µm.

In contrast to emulsion, Recovery Agent induced a strong CPE when directly added to the cells (Figure 2A) and, therefore, had to be removed from the virus solution. Since Recovery Agent clogged the Amicon size exclusion columns we usually use for removing toxic substances from virus solutions [14], we switched to Bio-Beads SM-2 resin because it can be used for batch purification [37]. Using this approach, it was possible to remove the toxic components of the Recovery Agent, enabling viral inactivation studies (Figure 2A). However, our data show that rVSV-GP_EBOV_-GFP was not inactivated when exposed to the Recovery Agent for 2 min, which is the contact time according to the Chromium sequencing protocol (Figure 2B). Mixing the virus solution with emulsion before adding the Recovery Agent did not lead to VSV inactivation either (Figure 2B). We, therefore, concluded that neither emulsion nor Recovery Agent can be used to chemically inactivate nonsegmented negative-sense RNA viruses.

### 3.3. Inactivation of Nipah Virus-Infected Cells Using Heat

Next, we tested whether heat inactivation could be used to inactivate NiV for scRNA-seq purposes. The first step in establishing this inactivation method was to identify a thermal cycler that allowed reliable sample inactivation using heat. We tested the Applied Biosystems™ MiniAmp™ Thermal Cycler (Figure S1A) because this instrument was already used in a BSL-3 setting for similar purposes [38]. To test the temperature reliability of the thermal cycler, an external digital temperature sensor (Figure S1B) was placed in a test tube filled with 125 μL of water, and the block temperature was set to 53 °C, 60 °C, or 85 °C and the temperature in the test tube was recorded. The test tube was also placed at different positions within the thermal cycler. The temperatures were chosen based on the scRNA-seq protocol that includes a 45 min incubation at 53 °C for reverse transcription followed by a 5 min incubation at 85 °C (see manual of the Chromium Next GEM Single Cell 3′ Reagent Kit v3.1). A 30-min incubation step at 60 °C was included as an additional heat inactivation step based on our previous data obtained with EBOV, showing that low numbers of EBOV-infected cells (less than 1 × 10^6^) can be inactivated under these conditions [9].

We did not observe temperature variations based on the position of the test tube within the thermal cycler (Figure S1C). In addition, the sample temperature matched the set temperature within the range of the expected accuracy of ±1 °C during the analyzed time intervals (Figure S1D). Taken together, our measurements show that the temperature settings of the MiniAmp thermal cycler are reliable. Therefore, this thermal cycler was used for the described inactivation studies.

We and others have previously shown that the precise conditions of heat-based viral inactivation are of utmost importance. Among other parameters, this includes cell numbers, viral load, temperature, and incubation time [14]. For this study, 6 × 10^4^ Vero E6 cells seeded in a 48-well plate were mock-infected or infected with NiV and 1 dpi, spun down, resuspended in 125 µL PBS, and transferred into 8-tube strips for heat inactivation in the thermal cycler. The mock-infected control sample was used to determine the cell number at the time of virus inactivation, which was 1 × 10^5^ cells per well. To determine the infection rate of the initial infection, an extra well was infected with NiV at the same MOI and used for immunofluorescence analysis. Virtually all cells were infected with NiV at 1 dpi (Figure S2).

**Figure 3:**
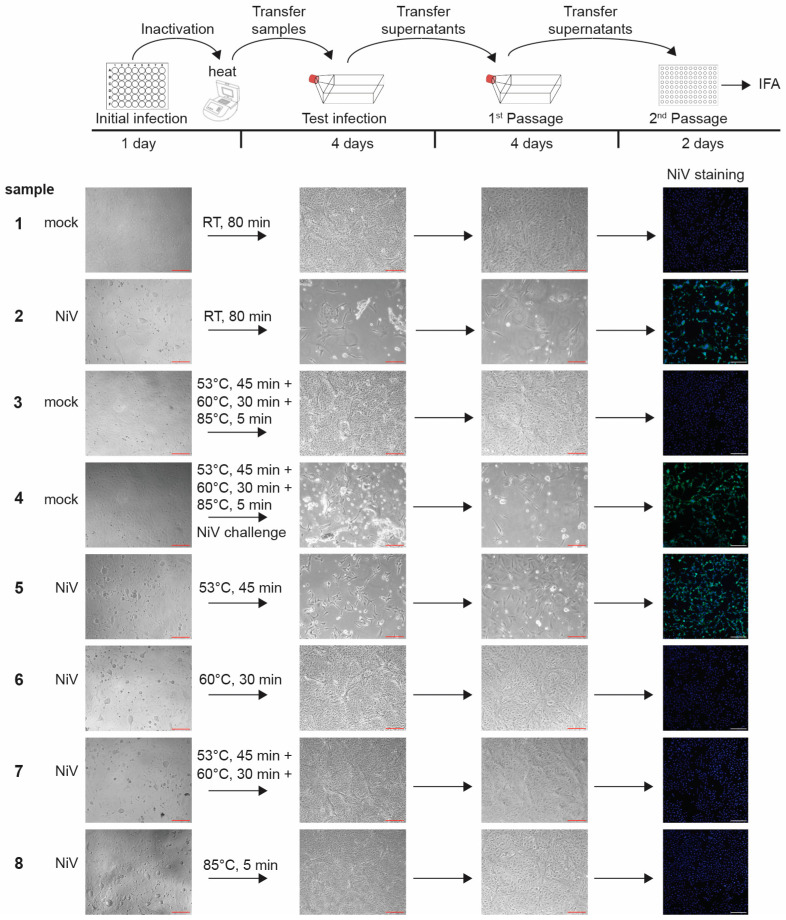
Nipah virus inactivation using heat. Top, schematic of the assay. Vero E6 cells seeded in 48-well plates were mock infected or infected with NiV at an MOI of 10. At 1 dpi, the NiV-infected cells showed clear cytopathic effects (CPE) and were harvested for inactivation (Initial infection). Cell pellets were resuspended in 125 µL of PBS, transferred into 8-tube strips, and exposed to the various heat steps in a thermal cycler as indicated. An 80 min incubation at room temperature was used as a negative control. The heat-treated cell lysates were then used to infect Vero E6 cells seeded in T75 flasks. Samples were monitored for NiV-induced CPE at 4 dpi (Test infection). Clarified cell supernatants were passaged onto Vero E6 cells seeded in T75 flasks. Cells were incubated for 4 days and monitored for CPE (1st Passage). Cell supernatants were then used to infect Vero E6 cells seeded in 96-well pates for immunofluorescence analysis using polyclonal anti-NiV mouse ascitic fluid (green; 2nd Passage). Cell nuclei were stained with DAPI (blue). Images represent two independent experiments with similar outcomes. Red scale bars 200 µm, white scale bars 250 µm.

After GEM formation, the reverse transcription step, which introduces single-cell barcoding, is carried out in a thermal cycler at 53 °C for 45 min, followed by an inactivation step at 85 °C for 5 min. Inactivating the viruses during or after reverse transcription assures that mRNA integrity is not compromised. We therefore tested both heat steps for their ability to inactivate NiV in addition to the extra heat step at 60 °C for 30 min (Figure 3). A NiV challenge sample was included to show that NiV can replicate in cells that were exposed to heat-treated cells. To do this, NiV was mixed with non-infected cells that had been exposed to all heat steps, and the mixture was used for infection (Figure 3, sample 4). The cells incubated at various conditions were then used to infect Vero E6 cells seeded in T75 flasks (Figure 3, Test infection). At 4 dpi, the entirety of the clarified cell supernatants was passaged onto fresh cells seeded in T75 flasks (Figure 3, 1st Passage). At 4 dpi, the supernatants from the 1st Passage were used to infect cells seeded in 96-well plates for immunofluorescence analysis using NiV-specific antibodies (Figure 3, 2nd Passage). All infections and passages were checked for signs of CPE.

Our data show that although the NiV infection rate was slightly diminished after an incubation time of 45 min at 53 °C, virus inactivation was incomplete (Figure 3, sample 5). In contrast, both individual incubation steps at 60 °C for 30 min and 85 °C for 5 min led to complete NiV inactivation at the chosen conditions (1 × 10^5^ infected cells; Figure 3, samples 6 and 8). We were not able to detect live NiV after these treatments, even after serial passaging. Not surprisingly, the combination of 53 °C for 45 min followed by 60 °C for 30 min also led to complete virus inactivation (Figure 3, sample 7). All the controls, including the NiV challenge sample, showed the expected outcome (Figure 3, samples 1–4).

Based on these data, our approved inactivation SOP consists of a 30 min incubation at 60 °C followed by a 5 min incubation at 85 °C for a maximum number of 1 × 10^5^ cells infected with NiV in a maximum volume of 125 µL. These inactivation steps are preceded by a 45-min incubation at 53 °C, which is required for cDNA synthesis but is not sufficient to inactivate NiV. As part of this inactivation SOP, the temperature within the thermal cycler must be measured using an external temperature sensor in addition to internal temperature control during inactivation.

### 3.4. GEM Formation Does Not Affect Heat Inactivation

**Figure 4:**
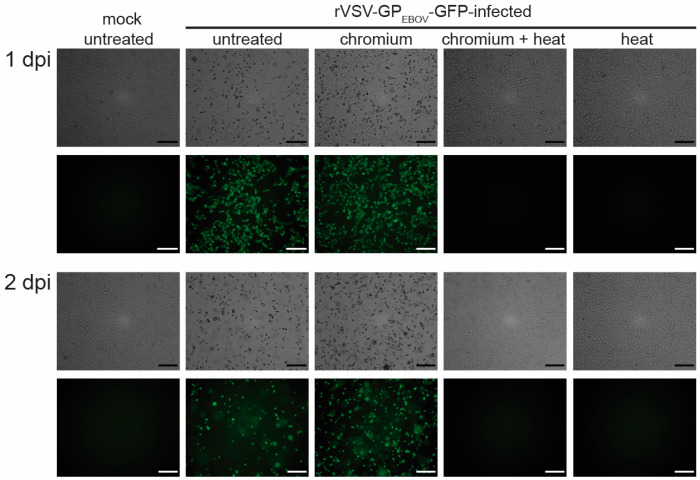
Processing samples using the 10x Genomics Chromium instrument (10x Genomics, Pleasanton, CA, USA) without heat treatment does not inactivate rVSV-GP_EBOV_-GFP. Vero E6 cells seeded in a 6-well plate were mock-infected or infected with rVSV-GP_EBOV_-GFP at an MOI of 1. 18 h post-infection, the cells were collected, and 1 × 10^5^ cells per sample were processed using the Chromium instrument with (chromium + heat) or without heat treatment (chromium) and used to inoculate Vero E6 cells seeded in T75 flasks. Controls included 1 × 10^5^ untreated mock-infected cells and 1 × 10^5^ untreated infected cells in 125 µL DMEM + 7% FBS that were used to inoculate Vero E6 cells seeded in T75 flasks. As a heat inactivation control, 1 × 10^5^ rVSV-GP_EBOV_-GFP-infected cells in 125 µL DMEM + 7% FBS were exposed to heat treatment without GEM formation (heat) and used to inoculate Vero E6 cells. Virus infection was monitored 1 and 2 dpi via GFP expression. Images represent two independent experiments with similar outcomes. Scale bars = 200 µm.

Next, we analyzed whether GEM formation in the Chromium microfluid chip would compromise heat inactivation of the samples. 1 × 10^5^ Vero E6 cells infected with rVSV-GP_EBOV_-GFP were used for GEM formation using the Chromium instrument according to the manufacturer’s instructions. One sample was subjected to cDNA synthesis, including the additional 30-min heat step at 60 °C, whereas the other sample was not exposed to heat after GEM formation. Controls included 1 × 10^5^ rVSV-GP_EBOV_-GFP-infected cells suspended in 125 µL DMEM + 7% FBS that were exposed to the heat steps (45 min at 53 °C; 30 min at 60 °C; 5 min at 85 °C). The samples were then used for inoculation of Vero E6 cells seeded in T75 flasks and incubated for 2 days. While we detected live viruses in the samples that were used for GEM formation without heat exposure, there was no detectable viral propagation in the cells infected with any of the samples exposed to heat (Figure 4). These data show that GEM formation in the Chromium instrument does not interfere with virus heat inactivation.

### 3.5. Limit of Detection Analysis for Nipah Virus

**Figure 5:**
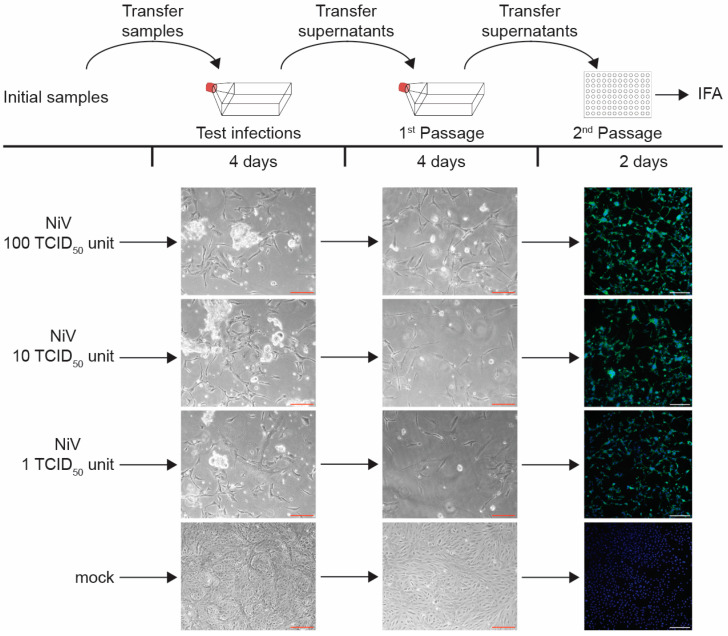
Limit of detection analysis for Nipah virus. Top, schematic of the assay. Vero E6 cells seeded in T75 flasks were mock infected or infected with NiV with the indicated TCID_50_ units (Test infections). At 4 dpi, clarified supernatants were passaged onto Vero E6 cells seeded in T75 flasks. Cells were incubated for 4 days and monitored for viral infection (1st Passage). Clarified supernatants were then used to infect Vero E6 cells seeded in 96-well plates and fixed at 2 dpi (2nd Passage). Immunofluorescence analysis was performed using polyclonal anti-NiV mouse ascitic fluid (green). Cell nuclei were stained with DAPI (blue). Images represent two independent experiments with similar outcomes. Red scale bars 200 µm, white scale bars 250 µm.

Our NiV heat inactivation analysis was accompanied by a limit of detection analysis to determine the sensitivity of our testing procedure. NiV stocks were diluted to the indicated virus amounts (1, 10, or 100 TCID_50_ units) and used to infect Vero E6 cells seeded in T75 flasks. The cell supernatants from these infections were passaged twice. The infected cells were checked for virus-induced CPE, and the second Passage was used for immunofluorescence analysis. There was visible CPE- and NiV-induced syncytia formation during the initial infection and subsequent passages for all virus amounts used (Figure 5). All samples identified as positive using CPE were also positive in the immunofluorescence analysis. These results show that our virus detection assays are sufficient to detect extremely small amounts of virus, consistent with our previous NiV inactivation studies, in which we reliably detected NiV infection in 4 out of 4 experiments using 10 TCID_50_ units as infectious dose and 2 out of 4 experiments using 1 TCID_50_ unit [14].

### 3.6. Heat Inactivation Does Not Affect Single-Cell RNA Sequencing Quality

To assess if adding a 30-min incubation time at 60 °C would affect the quality of downstream scRNA-seq results, we focused on iAT2 cells because we had already established a scRNA-seq pipeline for this cell type, which was utilized for this study. iAT2 cells at the air–liquid interface were dissociated, sorted, and processed for scRNA-seq on the Chromium instrument. During cDNA synthesis, the secondary heat step was added, with subsequent steps unchanged. Additionally, to mimic BSL-4 conditions in which samples are required to pass through MicroChem Plus before exiting the BSL-4 facility, one sample was submerged in a secondary container filled with 5% MicroChem Plus for 10 min prior to the addition of the Recovery Agent. Samples were then processed for quality control and profiled using scRNA-seq.

**Figure 6:**
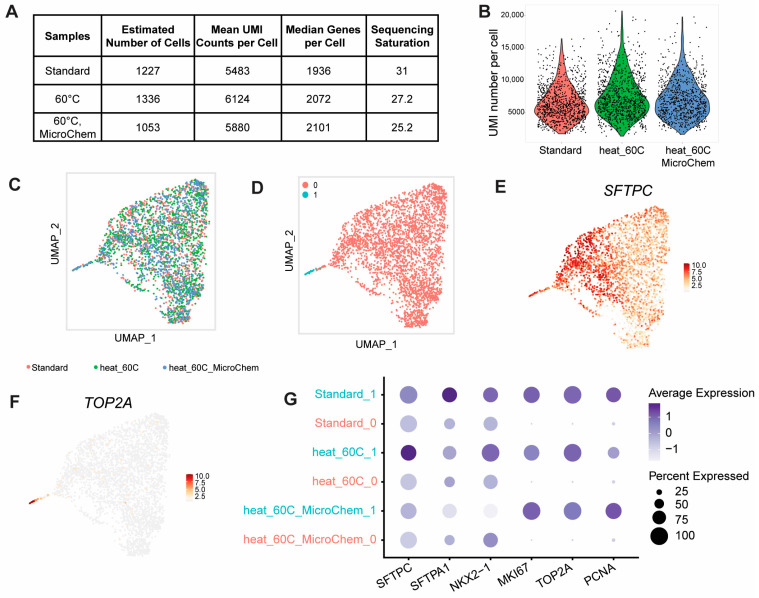
scRNA-seq analysis of iAT2 cells with and without an additional heat step. (**A**) Summary data of the three iAT2 samples processed for scRNA-seq. (**B**) Violin plot of unique molecular identifier (UMI) number per cell for each sample. (**C**) UMAP overlay of all samples. (**D**) Unsupervised Louvain clustering of the data. (**E**) Overlay of *SFTPC* expression. (**F**) Overlay of *TOP2A* expression. (**G**) Dot plot displaying expression of AT2 and proliferation markers across samples and clusters.

According to Bioanalyzer quality control, the resulting cDNAs from the three samples showed similar size distributions (Figure S3), and the sequencing libraries had the typical size distribution for 10x 3′ v3.1 library preparations. The estimated number of cells and mean unique molecular identifier (UMI) counts per cell were not significantly different across samples (Figure 6A,B). Knee plots of the samples revealed no noticeable differences, indicating that the extra heat step did not lead to more empty droplets or higher amounts of ambient RNA (Figure S4). We observed a similar percentage of mitochondrial content per cell in all three samples, indicating similar levels of cell integrity (Figure S5). By uniform manifold approximation and projection (UMAP), all three samples overlaid on top of each other (Figure 6C). The samples were transcriptionally very similar, as shown by unsupervised clustering, resulting in two clusters of cells (Figure 6D). Clusters 0 and 1 both expressed AT2 programs such as SFTPC (Figure 6E). The main difference of cluster 1 was that it presented the proliferating population as demonstrated using the upregulation of proliferation markers such as TOP2A (Figure 6F). Importantly, lung-related and AT2-related program markers *SFTPC*, *SFTPA1*, *ABCA3*, and *NKX2-1*, as well as proliferation markers *MKI67*, *TOP2A*, and *PCNA,* were expressed at similar levels across the three samples (Figure 6G). Comparing the transcriptional signatures of the samples, we observed no statistically significant differences between the heated sample (heat_60 °C) and the standard sample and only observed five differentially expressed genes enriched in the heated sample submerged in Microchem. This included three mitochondrial genes (MT-ND3, MT-ATP8, and MALAT1), one ribosomal protein gene (RPS29), and one non-coding RNA (PLCG2), each of which showed increased expression of less than 1.3-fold compared to the standard protocol sample. Overall, the transcriptomic results were highly similar across these settings, suggesting that the addition of the secondary heat step and the 10-min incubation in MicroChem Plus did not adversely affect RNA quality for scRNA-seq.

## 4. Discussion

The goal of this study was to establish a pipeline for the generation of BSL-4 scRNA-seq samples that both comply with the stringent biosafety requirements for BSL-4-derived samples and do not reduce sample quality or downstream scRNA-seq results. Regarding the work with BSL-4 agents, minimizing the time and workload within the BSL-4 space has high priority, and therefore, inactivating the samples at an early step of the sequencing procedure would be desirable. One possibility for generating BSL-4 scRNA-seq samples would be to inactivate the infected cells prior to microfluidic partitioning. This has been performed before using various fixatives, including methanol, 4% PFA, 10% formalin, including formalin-fixed paraffin-embedded (FFPE) tissues, and 3% glyoxal [6,7,39,40,41]. The major disadvantage to this approach is that the use of these fixatives generally results in lower-quality RNA in the samples, resulting in reduced library complexity [5,6,42]. Additionally, recent scRNA-seq studies using FFPE tissues focused on nuclear RNA sequencing and, therefore, lack the ability to detect cytoplasmic RNAs. For this reason, these approaches are ill-suited for the study of many virus-infected samples [43].

Another approach to generate BSL-4 scRNA-seq samples would be to generate the final DNA library for sequencing within the BSL-4 laboratory, inactivate the library using approved inactivation procedures for the isolation of nucleic acids, and remove the sample from the BSL-4 laboratory for further processing. This approach has the advantage of utilizing inactivation procedures that are already approved in most BSL-4 facilities, including Qiagen’s RLT buffer in combination with ethanol, Qiagen’s AVL buffer in combination with ethanol, TRIzol, or gamma irradiation, and is independent of the scRNA-seq approach [14,44,45,46,47,48,49]. However, it does involve increased time and work within the BSL-4. This approach is described in a companion article by Sturdevant, G. L. et al. [50].

To minimize time and workload within the BSL-4 space, we sought to identify intermediate steps during the scRNA-seq process—following the generation of single-cell suspensions and prior to full library preparation—that could serve to inactivate the virus. We focused on the 10x Genomics Chromium platform to prepare samples for scRNA-seq due to its ease of use, and we used NiV as our test virus because it is classified as a BSL-4 pathogen and Select Agent, which requires stringent inactivation conditions. We tested various chemical and physical components of the platform for their ability to inactivate NiV and found that many steps had no effect on virus viability. The only step in this process that was able to completely inactivate NiV was the 5 min incubation at 85 °C following the reverse transcription step. To satisfy the stricter biosafety regulations regarding BSL-4-derived samples, we added a heat step after cDNA synthesis and prior to the 85 °C step. This extra heat step of 30 min at 60 °C was sufficient to inactivate NiV-infected cells, adding a layer of redundancy to the inactivation of the virus during the modified Chromium protocol. Critically, the inclusion of this extra heat step did not alter sample quality or downstream scRNA-seq results. This approach has the advantage of limiting researcher time in BSL-4 but may require additional heat inactivation testing depending upon existing approved BSL-4 protocols.

Some of the work presented here was performed with VSV as a surrogate virus for nonsegmented negative-sense RNA viruses. Similar to NiV, 1 × 10^5^ cells infected with rVSV-GP_EBOV_-GFP were also rendered non-infectious when exposed to the two heat steps in our modified Chromium protocol: 30 min at 60 °C and 5 min at 85 °C. We are therefore confident that the proposed heat-based inactivation steps can be safely applied to other nonsegmented negative-sense RNA viruses as long as the described inactivation parameters, including maximum cell number and maximum sample volume, are strictly observed. Careful definition of heat inactivation parameters is paramount. We and others have previously shown that incomplete inactivation can occur when using larger sample volumes, higher amounts of infected cells or viruses, shorter incubation times, or lower temperatures, particularly those below 60 °C [9,14,44].

In conclusion, we have demonstrated that including an additional heat step (30 min at 60 °C) to the 10x Genomics Chromium sequencing protocol efficiently inactivates nonsegmented negative-sense RNA viruses, such as NiV and VSV, while not impacting RNA quality. This modified workflow enables the safe use of NiV-infected cells in scRNA-seq procedures and will inform scRNA-seq approaches on other highly pathogenic viruses.

## Data Availability

The data reported in this paper have been deposited in the Gene Expression Omnibus database under accession number GSE247406, and the reviewer token is kvstwisgrpsjdiv.

## Supplementary Materials

The following supporting information can be downloaded at: https://www.mdpi.com/article/10.3390/pathogens13010062/s1, Figure S1: Temperature measurements in in the Applied Biosystems™ MiniAmp™ Thermal Cycler. Figure S2: Initial NiV infection rates at one-day post-infection. Figure S3: cDNA quality of scRNA-seq samples. Figure S4: Knee plots of scRNA-seq samples. Figure S5: Scatter and violin plots.
