# Supplementary material for: Heat Inactivation of Nipah Virus for Downstream Single-Cell RNA Sequencing Does Not Interfere with Sample Quality
Source: Pathogens. 2024 Jan 9;13(1):62. doi: 10.3390/pathogens13010062 (PMC10818917; doi:10.3390/pathogens13010062)
Supplement: Supplementary file 1 [file pathogens-13-00062-s001.zip › pathogens-2776487-supplementary.pdf]

Supplementary Figures

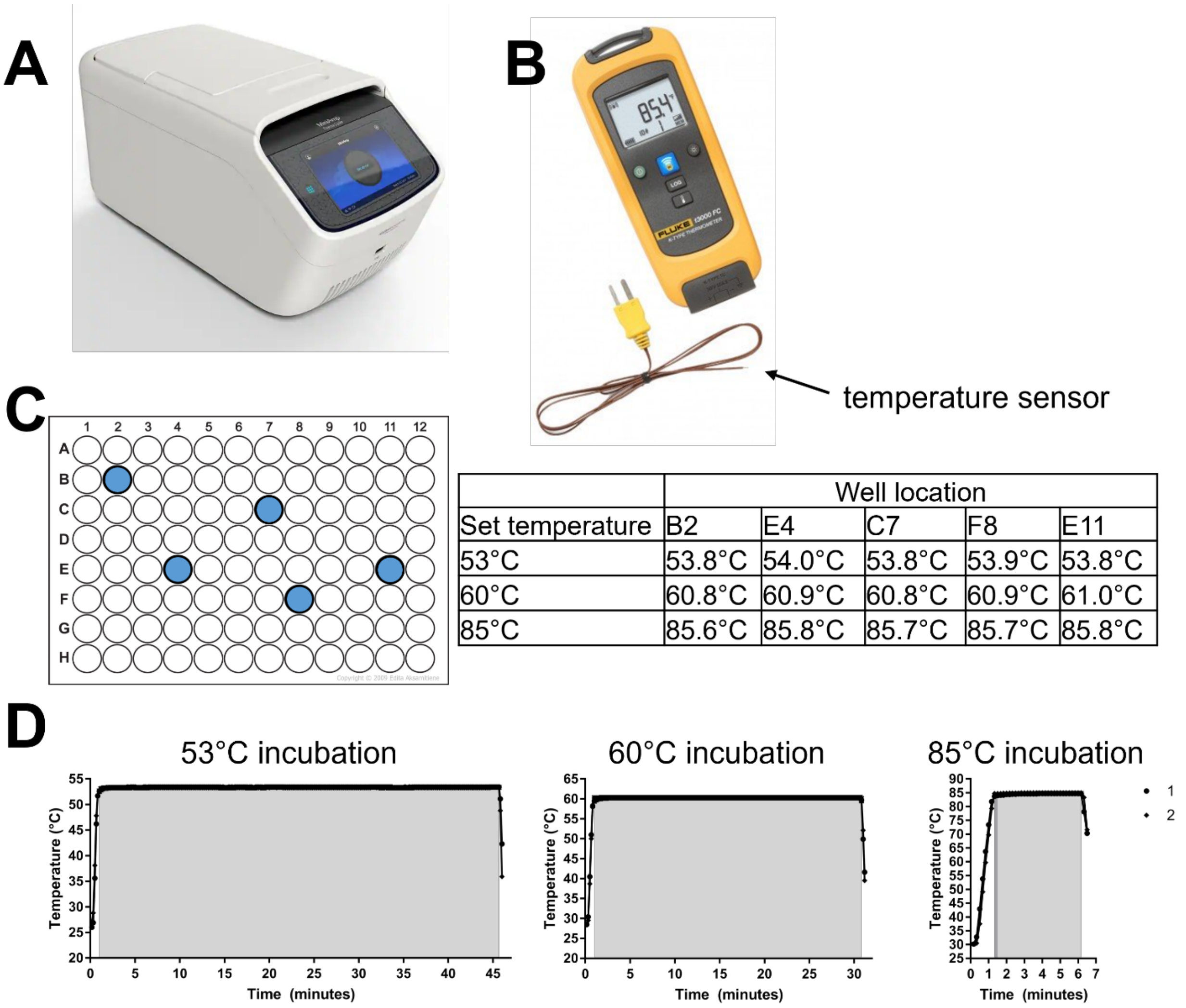

	Well location				
Set temperature	B2	E4	C7	F8	E11
53°C	53.8°C	54.0°C	53.8°C	53.9°C	53.8°C
60°C	60.8°C	60.9°C	60.8°C	60.9°C	61.0°C
85°C	85.6°C	85.8°C	85.7°C	85.7°C	85.8°C

Figure S1. Temperature measurements in the Applied Biosystems™ MiniAmp™ Thermal Cycler. (A) Image of the thermal cycler used for scRNA-seq sample inactivation in the BSL-4 laboratory. (B) Image of the Fluke T3000 FC Wireless K-Type Temperature Module. (C) Temperature measured with the Fluke T3000 FC Wireless K-Type Temperature Module at different positions in the thermal cycler. (D) Temperature profile measurements with the Fluke T3000 FC Wireless K-Type Temperature Module with thermal cycler set to programs of 53°C for 45 minutes (left), 60°C for 30 minutes (middle), and 85°C for 5 minutes (right) measured every 10 seconds. Measurements were performed twice (runs 1 and 2, as indicated by the symbols). Times in which the temperature probe measured within 1°C of the desired temperature are shaded in gray.

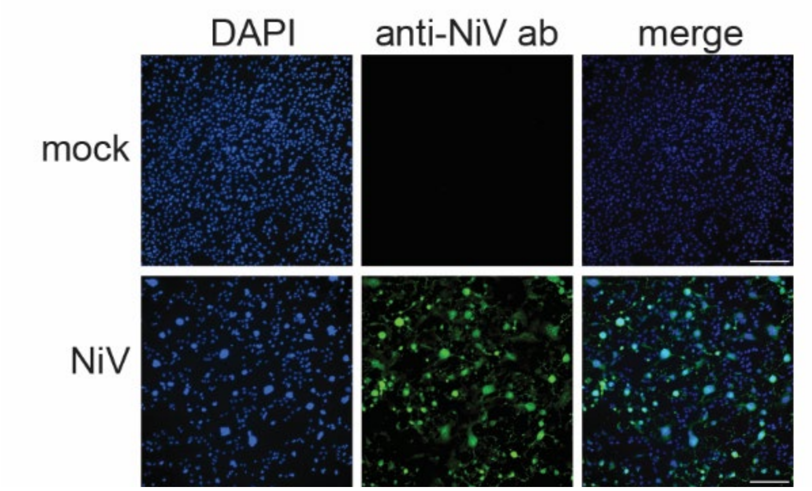

Figure S2. Initial NiV infection rates at one day post-infection. Vero E6 cells seeded in a 48-well plate were left uninfected (mock) or infected with NiV at a multiplicity of infection of 10 $TCID_{50}$ units per cell. One day post-infection, the cells were fixed with 10% formalin and subjected to immunofluorescence analysis using polyclonal anti-NiV hyperimmune mouse ascitic fluid (green). Cell nuclei were stained with DAP (blue).

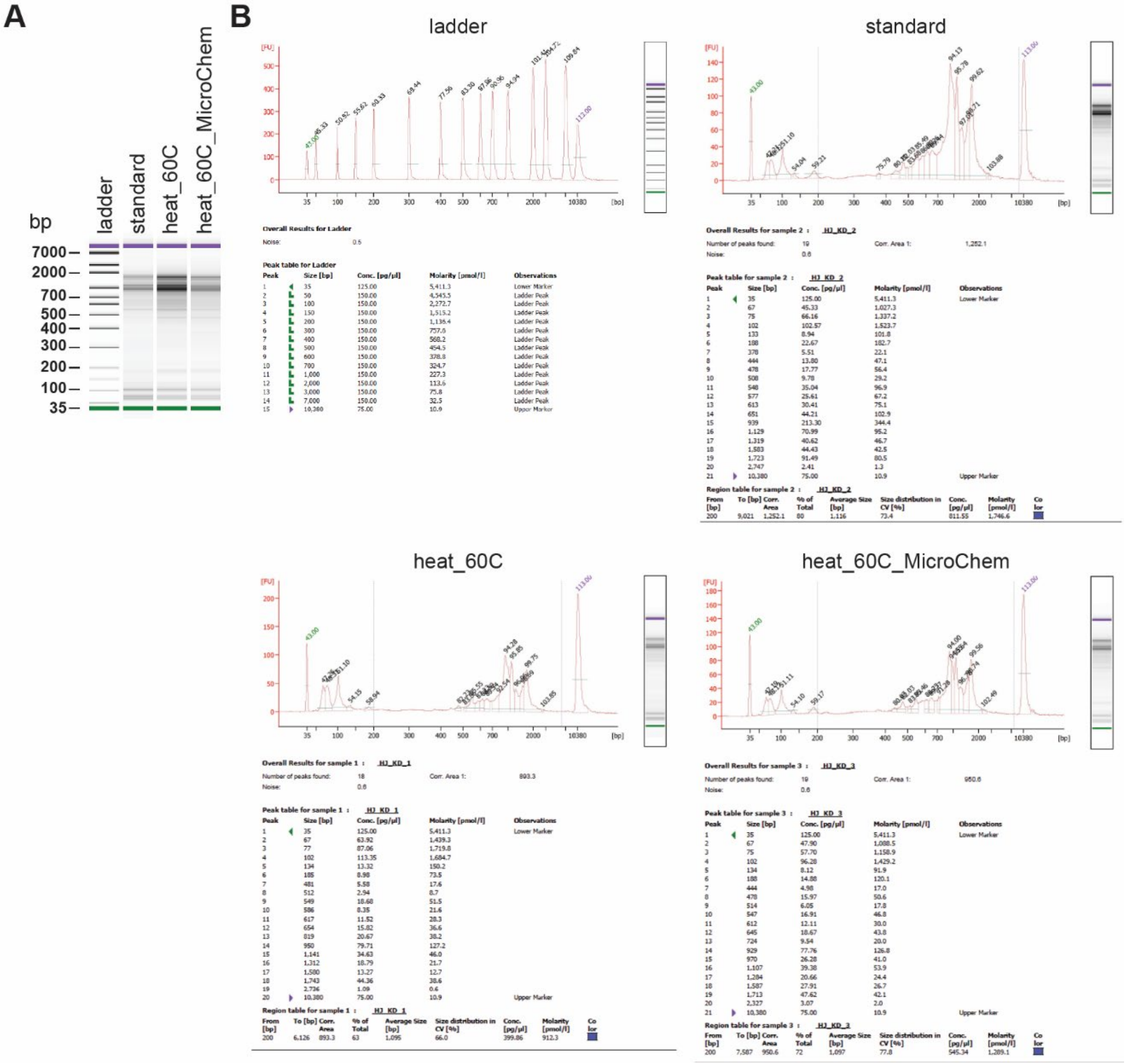

Figure S3. cDNA quality of scRNA-seq samples. (A) Bioanalyzer electropherogram of the generated cDNAs for each sample. (B) Electropherogram sample traces of cDNA for each of the three samples showing similar size distribution.

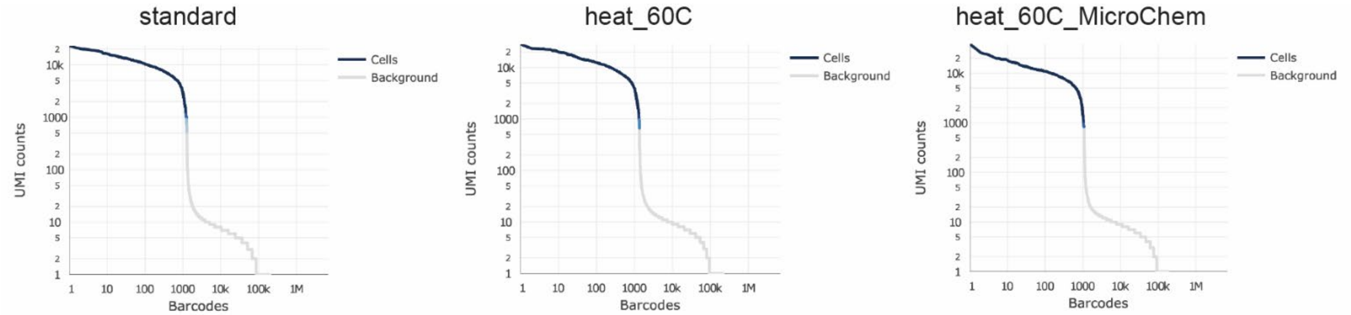

Figure S4. Knee plots of scRNA-seq samples. Knee plots of scRNA-seq of all three samples showing a steep drop-off separation between the cell-associated barcodes and the barcodes associated with empty GEMs. The percentage of reads mapped in cells was 93.4%, 93.3%, and 92.3% for the standard, heat_60C, and heat_60C_MicroChem samples, respectively.

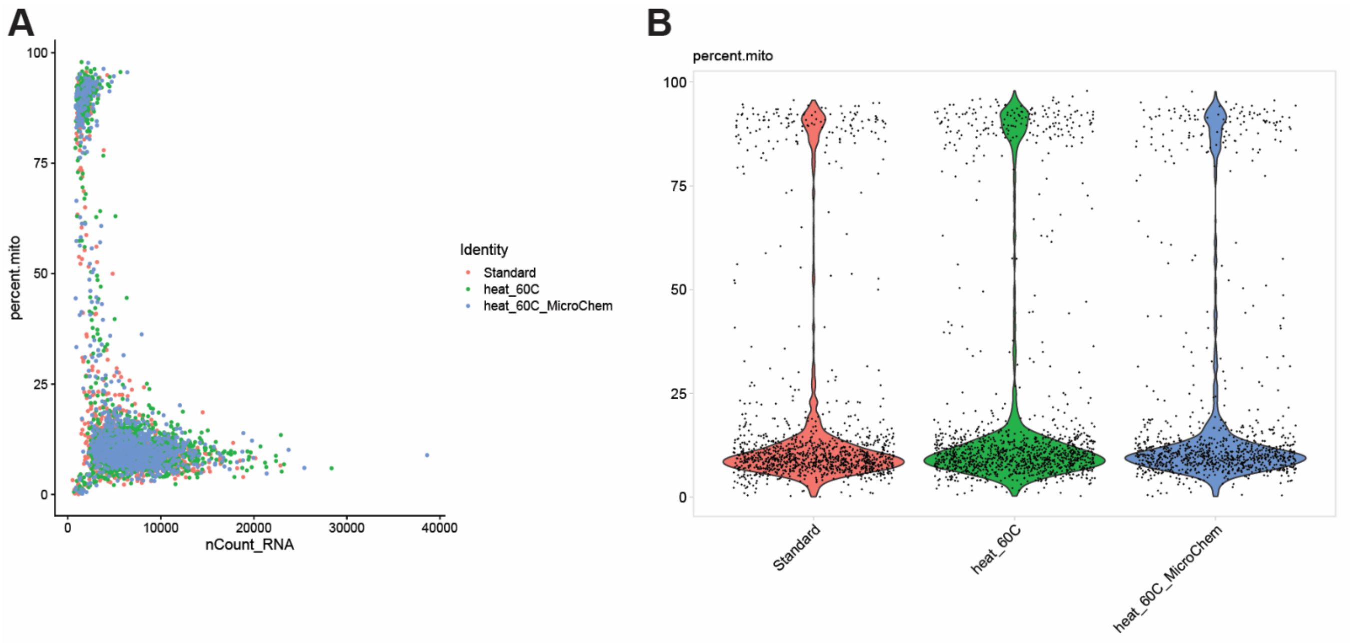

Figure S5. Scatter and violin plots. Scatter (A) and violin (B) plots showing percentage of mitochondrial transcripts per cell in all three scRNA-seq samples.
